# Bayesian Adaptive Extended Kalman-Based Orbit Determination for Optical Observation Satellites

**DOI:** 10.3390/s25082527

**Published:** 2025-04-17

**Authors:** Yang Guo, Qinghao Pang, Xianlong Yin, Xueshu Shi, Zhengxu Zhao, Jian Sun, Jinsheng Wang

**Affiliations:** 1 Shandong Key Laboratory of Space Debris Monitoring and Low-Orbit Satellite Networking, Qingdao University of Technology, Qingdao 266520, China; 2 Key Laboratory of Intelligent Space TT&O (Space Engineering University), Ministry of Education, Beijing 101416, China; 3 National Astronomical Observatories, Chinese Academy of Sciences, Beijing 100101, China; 4 Universe Kingdom Beijing Technology Co., Ltd., Beijing 100094, China

**Keywords:** orbit determination, optical observations, extended Kalman filter, Bayesian

## Abstract

As the number of satellites and amount of space debris in Low-Earth orbit (LEO) increase, high-precision orbit determination is crucial for ensuring the safe operation of spacecraft and maintaining space situational awareness. However, ground-based optical observations are constrained by limited arc-segment angular data and dynamic noise interference, and the traditional Extended Kalman Filter (EKF) struggles to meet the accuracy and robustness requirements in complex orbital environments. To address these challenges, this paper proposes a Bayesian Adaptive Extended Kalman Filter (BAEKF), which synergistically optimizes track determination through dynamic noise covariance adjustment and Bayesian a posteriori probability correction. Experiments demonstrate that the average root mean square error (RMSE) of BAEKF is reduced by 34.7% compared to the traditional EKF, effectively addressing EKF’s accuracy and stability issues in nonlinear systems. The RMSE values of UKF, RBFNN, and GPR also show improvement, providing a reliable solution for high-precision orbital determination using optical observation.

## 1. Introduction

In recent years, with the increasing frequency of space activities, more and more man-made spacecraft, such as satellites and rockets, have been sent into space orbit. These space activities are accompanied by the generation of large amounts of space debris. With the sharp increase in the number of space objects in orbit, the risk of collision between spacecraft and space objects has increased, and the space environment has become more hostile, posing great risks and obstacles to space activities [1]. This has led to an increasingly crowded and hostile space environment around the Earth, greatly increasing the risk of collision of spacecraft in orbit. In the face of all these threats, space science research on spacecraft protection has to be carried out. Satellite orbit determination has been a crucial research area in the development of modern space science and technology. Accurate orbit determination is critical not only for effective satellite operation and management, but also has important applications in space collision warning, scientific exploration missions, and national security [2,3,4]. Most traditional orbit-determination methods are based on accurate ranging and velocity data from ground-based observatories. However, in some cases, such as optical detection missions or resource-constrained scenarios, only angular observation data are available [5]. In this case, it becomes a challenge to realize high-precision orbit determination using limited optical angle observation data.

Accurate satellite orbits are the basis for providing users with high-precision services, and the determination of high-precision satellite orbits depends on the rational distribution and the number of Ground Tracking Stations (GTSs) [6]. Optical observation, as an important monitoring means, is widely used in satellite tracking due to its advantages of non-contact and all-weather compatibility, but its core challenge is that only short-arc observations of azimuth and elevation can be obtained. For example, in low-orbit space debris monitoring missions, optical telescopes are often limited by the observation time window, with a single observation lasting only a few minutes, resulting in multiplicity of orbital parameter solving, which seriously affects the accuracy of collision warning [7]. In addition, deep-space exploration missions require high-precision orbiting within a limited visible time window, and the dense deployment of micro-satellite constellations further requires the algorithms to converge quickly under sparse data conditions. Therefore, this study based on ground-based optical observations intends to address several difficulties faced by optical observations of spacecraft such as satellites. In addition, the BAEKF method proposed in this study is theoretically designed without limiting to a specific orbit type, and its adaptive noise covariance adjustment and Bayesian a posteriori correction mechanism can flexibly adapt to different setup dynamics-dominated environments. The current experimental validation is mainly based on LEO targets, whose dynamics model is dominated by the Earth’s flatness uptake and atmospheric drag. The orbital uptake of high-orbit satellites is dominated by solar and lunar gravitational forces, which requires adjusting the weights of higher-order terms in the dynamical model. Future research is needed to further validate the adaptability of BAEKF in high or deep-space orbits, especially for complex perturbation scenarios such as solar radiation and lunar gravitational uptake. First, spacecraft such as satellites are small in size themselves, and at the same time, they are mainly distributed in the Earth orbit, which is far away from the ground. In order to recognize and track the observation of space targets such as satellites, highly sensitive optical equipment and complex image processing techniques are required [8]. Second, optical observations are usually accompanied by disturbing factors such as atmospheric disturbances, cloud obscuration, stellar interference, optical process, or signal transmission problems, which may lead to shorter duration of continuous and effective observations [9,10]. Third, optical telescope observations alone can provide only angular observation information. Therefore, studies need to rely on a small amount of angular observation information to make the determination of the satellite orbit.

For the short-arc data of optical angle-only observations, this paper proposes a Bayesian Adaptive Extended Kalman Filter method to realize the initial satellite orbit determination using angle-only observation data [11]. Therefore, an adaptive factor is added to the EKF, which is used to improve the model’s ability to handle noise. At the same time, a Bayesian approach is introduced to give probability to the state estimation by processing the probability distribution of the system state and the model’s ability to handle nonlinear systems. The improved BAEKF reduces the root mean square error (RMSE) of the position by 32.6% compared to the conventional EKF, the fluctuation of the residuals by about 40%, and the average RMSE by 34.7%.

### 1.1. Related Work

In recent years, nonlinear filtering and Bayesian methods have made significant progress in the field of orbit determination, but there are still many challenges in applying them to optical short-arc observations [12,13]. Space-based target surveillance equipment can provide effective surveillance of deep-space targets due to its location, and space-based detection equipment is not affected by turbulence in the Earth’s atmosphere and does not require additional adaptive optics to correct optical aberrations. In addition, space-based target surveillance equipment possesses no geographical, meteorological, or time-of-use limitations [14]. Compared with space-based observatories, ground-based observatories, because they are not limited by space, weight, and size, can use large-diameter optical lenses to obtain high spatial resolution, and in the case of radars, the transmitter power can be increased to achieve longer detection ranges. Aarnes et al. [15] approximated the nonlinear distribution by approximating the Unscented Kalman’s traceless transform, which reduces the linearization error, but the computational complexity grows cubically with the state dimension, making it difficult to meet the real-time requirements. Gong et al. [16] solved the angular orbit-only observability problem by introducing a radial basis function neural network (RBFNN) to capture the nonlinearity of the perturbation dynamics, but the network training relies on a large number of historical data, which makes it difficult to adapt to sudden noise disturbances. Hou et al. [17] proposed a short-arc joint orbiting algorithm by enhancing the convergence speed through geometrical constraints through joint optical–optical observations on the ground and in space, but did not address the time-varying problem of noise. Sun et al. [18] developed a novel fast detection and orbit parameter determination method based on cooperative observation from space stations, and developed a fly-by-wire nanosatellite to determine the orbit parameters of a non-cooperative target using an Unscented Kalman Filtering method. Lee et al. [19] proposed and analyzed a strategy to correlate multiple orbital solutions using an optical spatial surveillance system and obtain an accurate orbital solution for predicting long-term trajectories, and verified the practical applicability by applying real tracking data from OWL-Net (Optical Wide-field Patrol Network). David et al. [20] used the GPR (Gaussian Process Regression) method to realize purely angular initial orbit determination, but it is highly sensitive to the orbit geometry and the computation grows exponentially with the number of data. Currently, many other scholars have investigated the use of Gaussian hybrid filters for orbit determination under conditions such as angle-only data [21,22,23,24].

### 1.2. Contributions in This Article

The conventional Extended Kalman Filter approximates nonlinear systems by local linearization and performs well in weakly nonlinear scenarios, but its inherent shortcomings are gradually exposed in optical observation applications. First, EKF’s neglect of higher-order terms in the nonlinear dynamical model leads to the accumulation of linearization errors, and the bias is especially significant in long-period orbit prediction. Second, EKF’s RMSE in position can be on the order of kilometers compared to real orbits for short-arc data with only angular observations. Third, EKF assumes that the statistical properties of the process noise and observation noise are fixed, which makes it difficult to cope with dynamically changing noise in real environments. Finally, EKF is highly sensitive to the initial state and is prone to divergence due to initial error amplification when data are sparse. To address the above problems, this paper proposes an optical observation orbit determination method based on a Bayesian Adaptive Extended Kalman Filter, which is innovative in the following three aspects:

1. Dynamic noise covariance adjustment: The process noise covariance matrix $Q_{k}$ and observation noise covariance matrix $R_{k}$ are optimized online through adaptive factors to adapt to environmental noise fluctuations in real-time. When the observation error increases due to sudden atmospheric disturbances, BAEKF can automatically reduce the weight of the anomalous data to avoid filter dispersion.
2. Bayesian a posteriori probability correction: A Bayesian inference framework is introduced to combine the a priori distribution with real-time observation to update the a posteriori probability distribution of the state estimation, effectively suppressing the linearization error accumulation. Experiments show that this method improves the position estimation accuracy by more than 30% compared with EKF in strongly nonlinear scenarios.
3. Fast convergence with short-arc data: By fusing geometric constraints from multi-moment angular observations, BAEKF can achieve stable convergence of orbital parameters in a short time.

### 1.3. Organization of the Paper

The paper is organized as follows. In Section 2, the paper describes the establishment of the relevant models, including the orbit dynamics model and the celestial angle measurement model. In Section 3, the method of calculating the position vector and velocity vector using the optical angle observation data is explained. Section 4 describes the principle of the Extended Kalman Filter and the principle of the Bayesian Adaptive Extended Kalman Filter proposed in this study. Section 5 demonstrates the observed object data, including parameters such as observation time, azimuth angle, elevation angle, etc. In Section 6, the proposed method is experimentally tested and the results are analyzed. Finally, in Section 7, the conclusion of this study is drawn and the direction of subsequent work is discussed.

## 2. Kinematic and Dynamical Model

### 2.1. Coordinate System

**Figure 1:**
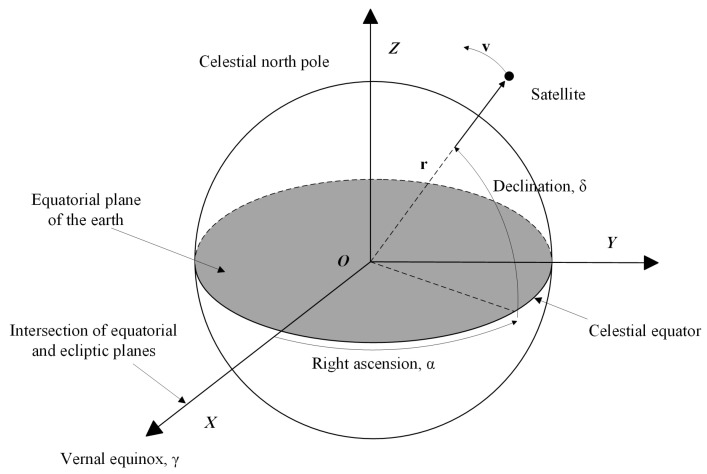
Definition of Earth-Centered Inertial coordinate system.

Since measurement models and motion models of space objects are built based on coordinate systems, defining a suitable system can make the corresponding problems easier to solve and reduce the amount of computation [1]. As shown in Figure 1, the Earth-Centered Inertial (ECI) astronomical coordinate system is used for the observations and calculations in this paper, which has the origin O at the center of the Earth, and its reference system remains fixed and does not change with the rotation of the Earth. The X-axis of this coordinate system points in the direction of the vernal equinox, the intersection of the celestial equator with the ecliptic plane. The Y-axis of the coordinate system forms a right-handed coordinate system with the X- and Z-axes, with the Y-axis in the equatorial plane and perpendicular to the X-axis.

### 2.2. Spatial Dynamics Modeling

In the ECI coordinate system, the position vector and velocity vector of the observed satellite are defined as r and v, respectively, with the following expressions:

(1) $$r={\left(\begin{array}{ccc} x & y & z \end{array}\right)}^{T}$$

(2) $$v=\dot{r}={\left(\begin{array}{ccc} v_{x} & v_{y} & v_{z} \end{array}\right)}^{T}+w_{v}$$

where x, y, and z, respectively, are the position components of the position vector in the ECI coordinate system. $v_{x}$, $v_{y}$, and $v_{z}$ are the velocity components of the velocity vector v in the ECI coordinate system, respectively. $w_{v}$ is the process noise term for the velocity. Considering the three-body problem of the Earth, the Moon, and the satellite, the acceleration of the satellite’s motion is jointly acted by the Earth’s gravity, the Moon’s gravity, and the regenerative force, which can be specifically expressed as the second-order derivative of the position, as shown in the following equation:

(3) $$a=\ddot{r}=-\mu_{e}\frac{r}{{\left|r\right|}^{3}}-\mu_{M}\frac{r-r_{M}}{|r-r_{M}{|}^{3}}+a_{perturb}$$

where $\mu_{e}$ and $\mu_{M}$ are the standard gravitational parameters of the Earth and the Moon, respectively, $\mu_{e}=Gm_{e}$ and $\mu_{M}=Gm_{M}$; *G* is the gravitational constant, $m_{e}$ is the mass of the Earth, and $m_{M}$ is the mass of the Moon; $r_{M}$ is the position vector of the moon; $a_{perturb}$ is the satellite acceleration caused by the sum of various perturbing forces; the main perturbing forces include Earth’s oblateness, Earth’s atmospheric drag, the pressure due to solar radiation, and the pressure due to solar radiation. Therefore, the state vector and state model of the spatial target are, respectively, the following:

(4) $$X={\left[r^{T}v^{T}\right]}^{T}$$

(5) $$\dot{X}=\left[\begin{array}{c} v\\ a \end{array}\right]=\left[\begin{array}{c} v_{x}+w_{vx}\\ v_{y}+w_{vy}\\ v_{z}+w_{vz}\\ -\mu_{e}\frac{x}{{\left|r\right|}^{3}}-\mu_{M}\frac{x-x_{M}}{|r-r_{M}{|}^{3}}+a_{x-perturb}\\ -\mu_{e}\frac{y}{{\left|r\right|}^{3}}-\mu_{M}\frac{y-y_{M}}{|r-r_{M}{|}^{3}}+a_{y-perturb}\\ -\mu_{e}\frac{z}{{\left|r\right|}^{3}}-\mu_{M}\frac{z-z_{M}}{|r-r_{M}{|}^{3}}+a_{z-perturb} \end{array}\right]$$

Denote the state transfer function by $f(X_{k})$ and the covariance matrix by $Q_{k}$ with $W_{k}$ as the state model process noise. Ultimately, this nonlinear motion system can be represented by the state transfer equation as follows:

(6) $${\hat{X}}_{k+1}=f\left(X_{k}\right)+W_{k}$$

### 2.3. Celestial Angle Observation Model

In optical astronomical observations, angular measurement models of celestial objects are used to convert images of celestial objects recorded by observing equipment into angular parameters such as azimuth and elevation. Azimuth is the angle clockwise from north to the projection of the celestial body on the horizontal plane, and elevation is the angle upward from the horizontal plane to the celestial body. The observation equations for the relevant parameters are as follows:

(7) $$Az=\arctan\left(\frac{y-y_{R}}{x-x_{R}}\right)$$

(8) $$El=arcsin\left(\frac{z-z_{R}}{\sqrt{{\left(x-x_{R}\right)}^{2}+{\left(y-y_{R}\right)}^{2}+{\left(z-z_{R}\right)}^{2}}}\right)$$

Set the location coordinates R of the ground-based optical observatory to $\left(x_{R},y_{R},z_{R}\right)$ and the location coordinates r of the celestial body to (x,y,z). The vector L from the position of the observatory to the position of the object and the distance ρ between the two can be expressed as follows:

(9) $$L=r-R=\left[\begin{array}{c} x-x_{R}\\ y-y_{R}\\ z-z_{R} \end{array}\right]$$

(10) $$\rho =\sqrt{{\left(x-x_{R}\right)}^{2}+{\left(y-y_{R}\right)}^{2}+{\left(z-z_{R}\right)}^{2}}$$

The $h(X_{k})$ denotes the observation function. At the same time, the observation noise $V_{k}$ is introduced with a covariance matrix of $R_{k}$ due to inevitable measurement error or noise in the actual observation. The observation noise reflects the observation error caused by the environment, the performance of observation equipment, and other factors. By modeling the observation noise, the error can be handled in filtering or optimization calculations, thus improving the accuracy of orbit estimation. The observation model can be expressed as follows:

(11) $$Z_{k}=h\left(X_{k}\right)+V_{k}$$

## 3. Position and Velocity Estimation

**Figure 2:**
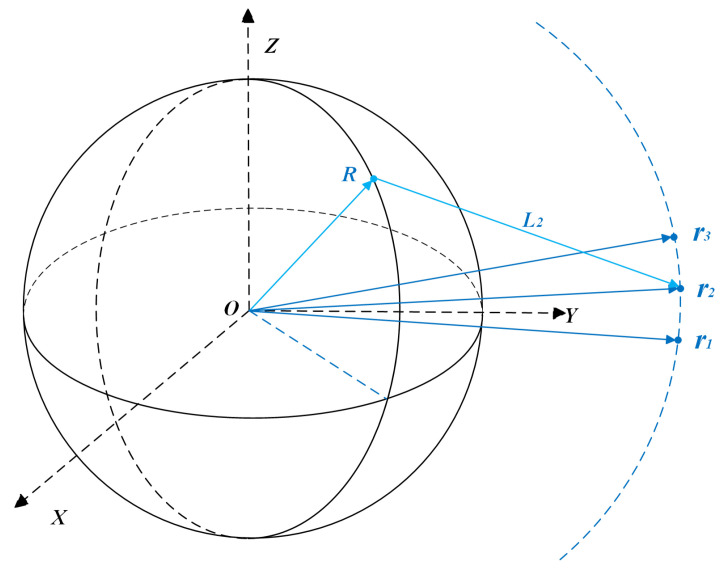
Multiple angles to calculate the celestial body position vector and velocity vector.

Ground-based optical observations face greater challenges in the orbit determination process because they can only obtain angular observation information, while distance information cannot be measured. In order to respond to this difficulty, it is necessary to calculate the distance between the observed satellite and the ground-based observatory by using more angular information to obtain the coordinates of the satellite’s position in the ECI coordinate system. As shown in Figure 2, R denotes the position coordinates of the ground-based optical observatory; $r_{i}$ denotes the position vectors optimized iteratively by the Newton–Raphson method at different observation moments; and $L_{2}$ denotes the distance estimate. First, a preliminary estimation of the orbit parameters is needed to calculate the position vector and velocity vector of the satellite at the current moment, and then the preliminary estimated orbit parameters are optimized using the Bayesian Adaptive Extended Kalman Filter theory.

### 3.1. Position Vector Estimation

Assuming that the study uses angular observations at three different moments $\left(t_{1},t_{2},t_{3}\right)$, the azimuth and elevation angles at the time of observation can be converted to right ascension $\left(\alpha_{1},\alpha_{2},\alpha_{3}\right)$ and declination $\left(\delta_{1},\delta_{2},\delta_{3}\right)$ using the geographic coordinates of the ground-based optical stations, the elevation data at the observation sites, and the time of observation. Convert three moments of angular observations into unit direction vectors $\left(l_{1},l_{2},l_{3}\right)$. The formula for the unit direction vector is as follows:

(12) $$l_{i}=\left(\begin{array}{c} \cos\left(\delta_{i}\right)\cos\left(\alpha_{i}\right)\\ \cos\left(\delta_{i}\right)\sin\left(\alpha_{i}\right)\\ \sin(\delta_{i}) \end{array}\right),\;i=1,2,3$$

An initial distance ρ is set, and the celestial position vectors for the three observation moments are initially estimated from the set initial distance and direction vectors. Using the iterative Newton–Raphson method, the observed direction vectors and the estimated position vectors are made to match more closely by continuously adjusting the initial distance ρ. In each iteration step, the distance estimate is adjusted using the update formula. The specific formula is as follows:

(13) $$L_{i}=\rho_{0}l_{i},\;i=1,2,3$$

(14) $$\rho^{\left(k+1\right)}=\rho^{\left(k\right)}-\frac{f(\rho^{\left(k\right)})}{f^{\prime }\left(\rho^{\left(k\right)}\right)}$$

where $f\left(\rho \right)=|\rho l_{i}-L_{i}|$ is the error function, representing the difference between the current estimated distance and the actual observations, and $f^{\prime }\left(\rho \right)$ is its derivative. The accurate celestial position vector L is calculated by iteratively optimizing the finalized distance ρ. Meanwhile, combining with Figure 2, the azimuth and elevation data of the three observation moments are converted to the unit direction vector $l_{i}$ via Equation (12), which is combined with the station position R and the initial distance estimation $\rho_{0}$ to initially calculate the satellite position. The geometric error is minimized by the iterative optimization ρ of the Newton–Raphson method, and the accurate satellite position vector is finally determined. This process provides the BAEKF with an initial state estimate $X_{0}$ whose uncertainty is characterized by the covariance matrix $P_{0}$.

### 3.2. Velocity Vector Estimation

With the time data and position data available, the study can calculate the initial velocity of the object at the moment of observation. The time interval τ between two neighboring observation moments and the position vector r of the observation moments are calculated, respectively. The specific calculation formula is as follows:

(15) $$\tau_{1}=t_{2}-t_{1}$$

(16) $$\tau_{3}=t_{3}-t_{2}$$

(17) $$r_{i}=l_{i}+R\;i=1,2,3$$

After obtaining the time intervals τ of the different observations and the position vectors r of the observations, Lagrange interpolation is used to compute the weighting coefficients D of the celestial velocities as well as the velocity vectors $v_{2}$ of the celestial bodies at the intermediate moments. The specific calculation formula is as follows:

(18) $$\left.\left\{\begin{array}{c} D_{1}=-\frac{\tau_{3}}{\tau_{1}\left(\tau_{1}+\tau_{3}\right)}\\ D_{3}=\frac{\tau_{1}}{\tau_{3}\left(\tau_{1}+\tau_{3}\right)}\\ D_{2}=1-D_{1}-D_{3} \end{array}\right.\right.$$

(19) $$v_{2}=D_{1}r_{1}+D_{2}r_{2}+D_{3}r_{3}$$

These weighting coefficients are used to linearly combine the observations to estimate the velocity of the object at intermediate observation moments. This velocity estimate is used as an initial input to the BAEKF, and its errors are gradually converged by adaptive noise covariance adjustment and Bayesian a posteriori correction to improve the accuracy and robustness of the orbit determination, and ultimately output the optimized orbit parameters.

## 4. Design of Extended Kalman Filter

The Extended Kalman Filter (EKF) is a derivative of the Kalman filter designed to solve the problem of state estimation for nonlinear systems [25]. The basic idea is to perform a first-order Taylor expansion of the system by neglecting the higher-order terms, ignoring the second-order and other terms above the second order, assuming that the linearized state still satisfies the Gaussian distribution, and transforming the nonlinear problem into a linear one, thus applying the framework of Kalman filter [26]. In EKF, the nonlinear state transfer function and the observation function are linearized into their respective Jacobi matrices to approximate the behavior of the nonlinear system by local linearization [27]. However, when applied to strongly nonlinear systems, the filtering may suffer from dissipation. The state of the celestial motion trajectory can be represented by the discrete system equations, and its state model and observation model can be expressed as follows:

(20) $$\left\{\begin{array}{c} X_{k+1}=f\left(X_{k}\right)+W_{k}\\ Z_{k}=h\left(X_{k}\right)+V_{k} \end{array}\right.$$

### 4.1. Initial State Estimation

When initializing the state vector, the initial state estimate ${\hat{X}}_{\left(0|0\right)}$ is first set, containing the initial estimates of position and velocity. Also, in order to represent the uncertainty of the initial state, the initialized state covariance matrix $P_{\left(0|0\right)}$ is introduced. The covariance matrix reflects confidence in the initial state estimation, with larger covariance values indicating greater uncertainty and smaller covariance values indicating higher confidence. The setting of the initial covariance matrix ensures that the filter can effectively perform state estimation and uncertainty updating in the initial stage. The specific formula for the initial state is as follows:

(21) $${\hat{X}}_{\left(0|0\right)}=X_{0}$$

(22) $$P_{\left(0|0\right)}=P_{0}$$

### 4.2. Forecasting Steps

The prediction step of Extended Kalman can be roughly divided into the following three steps:

First, the state ${\hat{X}}_{\left(k|k-1\right)}$ at the current moment is predicted by the state transfer equation based on the state estimation and the system model ${\hat{X}}_{\left(k-1|k-1\right)}$ is at the previous moment. Second, by calculating the partial derivatives of the state transfer function with respect to the state vectors, the Jacobi matrix $F_{k}$ is obtained, which can be used to linearize the model of the nonlinear system, enabling the filter to be computed efficiently on the nonlinear system.

Third, the partial derivatives of the observation function *h* with respect to the state vector X are computed to obtain the Jacobian matrix of the observation matrix $H_{k}$. The Jacobian matrix $H_{k}$ is used to linearize the nonlinear observation model, thus facilitating the subsequent Kalman gain computation and update steps. Finally, the covariance matrix $P_{\left(k|k-1\right)}$ at the current moment is predicted, which reflects the change in uncertainty of the state estimate during propagation. The specific calculation formula is as follows:

(23) $${\hat{X}}_{\left(k|k-1\right)}=f\left({\hat{X}}_{k-1|k-1}\right)+W_{k}$$

(24) $$F_{k}=\frac{\partial f}{\partial X}|{\hat{X}}_{k-1|k-1}$$

(25) $$H_{k}=\frac{\partial h}{\partial X}|{\hat{X}}_{k|k-1}$$

(26) $$P_{k|k-1}=F_{k}P_{k-1|k-1}F_{k}^{T}+Q_{k}$$

where $Q_{k}$ is the process noise covariance matrix, which represents the influence of uncontrollable factors in the system dynamics. By extending Kalman’s prediction step, the system is able to predict the target state at each moment and propagate the state uncertainty to the next moment.

### 4.3. Updating Steps

The update step performs a state update by combining observations while adjusting the noise covariance matrix based on the observation residuals. The observation residual $y_{k}$ is the difference between the observed and predicted observations at the current moment and is used to quantify the deviation between predicted and actual observations [28]. The residuals are an important basis for updating the state estimates, and the predicted states are corrected by the residuals to improve the estimation accuracy. Also, in order to balance the weights of state prediction error and observation error in the updating process, it is necessary to compute the observation covariance $S_{k}$, which is both the covariance matrix of the observation residuals and reflects the uncertainty of the observation residuals. In order to weigh the prediction error against the observation error, it is necessary to calculate the Kalman gain $K_{k}$, which determines the extent to which the observations influence the state estimate correction. The specific calculation formula is given below:

(27) $$y_{k}=Z_{k}-h\left({\hat{X}}_{k|k-1}\right)$$

(28) $$S_{k}=H_{k}P_{k|k-1}H_{k}^{T}+R_{k}$$

(29) $$K_{k}=P_{k|k-1}H_{k}^{T}S_{k}^{-1}$$

The state estimate is obtained by weighting the predicted state with the corrected state of the observed data ${\hat{X}}_{\left(k|k\right)}$. The updated covariance matrix ${\hat{P}}_{\left(k|k\right)}$ reflects the uncertainty correction of the state estimate. The specific calculation formula is as follows:

(30) $${\hat{X}}_{k|k}={\hat{X}}_{k|k-1}+K_{k}y_{k}$$

(31) $$P_{k|k}=\left(I-K_{k}H_{k}\right)P_{k|k-1}$$

The updating process enables each filtering iteration to gradually improve the estimation accuracy and reduce the estimation uncertainty, achieving an accurate estimation of the system state finally.

## 5. Design of Adaptive Extended Kalman Filter

The conventional EKF performs well in dealing with systems with fixed noise characteristics and can provide accurate estimates. However, when facing dynamically changing noise environments, the EKF is unable to adaptively adjust the covariance matrices of the process noise and the observation noise, which leads to a deterioration in its ability to adapt to changes in noise, thus triggering a decrease in filtering accuracy [29,30]. Especially in highly nonlinear systems, the linearization process of the EKF may introduce large errors, which in turn affect the accuracy and stability of the state estimation.

In addition, although EKF has low computational complexity in real-time data processing, frequent linearization operations in complex nonlinear systems tend to increase the computational burden, which in turn reduces the computational efficiency of the system. In order to solve the problems arising from the application of EKF, this study introduces the Bayesian method and adaptive tuning mechanism, aiming to improve the adaptability and robustness of the system in dynamic environments. With the Bayesian framework, the EKF is able to handle uncertainties in nonlinear systems more effectively, thus improving the accuracy of state estimation and demonstrating greater robustness and computational efficiency in complex nonlinear application scenarios such as orbit determination. Meanwhile, the introduction of the adaptive adjustment mechanism further enhances the adaptive ability of EKF to the dynamic noise environment, enabling the filter to adjust the noise covariance matrix in real-time to adapt to different noise characteristics and environmental changes, and to improve the performance and stability of the EKF in real complex systems.

### 5.1. Bayesian Reasoning

The core of Bayesian inference is to improve the accuracy of parameter updates by using observations to update the prior distribution to obtain the posterior distribution of the parameters. In the Bayesian framework, the prior distribution reflects initial assumptions or prior knowledge about the parameters, and by continuously obtaining new observations, the prior distribution can be revised to obtain a more accurate posterior distribution [31]. First, define the prior distribution, assuming that α and β obey the Beta distribution, respectively, and their probability density functions can be expressed as follows:

(32) $$p\left(\alpha \right)=Beta\left(\alpha ;a_{\alpha },b_{\alpha }\right)=\frac{\alpha^{a_{\alpha }-1}{\left(1-\alpha \right)}^{b_{\alpha }-1}}{B(a_{\alpha },b_{\alpha })}$$

(33) $$p\left(\beta \right)=Beta\left(\beta ;a_{\beta },b_{\beta }\right)=\frac{\beta^{a_{\beta }-1}{\left(1-\beta \right)}^{b_{\beta }-1}}{B(a_{\beta },b_{\beta })}$$

where $a_{\alpha }$, $b_{\alpha }$, $a_{\beta }$, and $b_{\beta }$ are the parameters of the prior distribution, which can be estimated using the maximum likelihood estimation (MLE) method when preliminary observations are available. In the above, the Beata distribution is chosen as the prior distribution for the parameters α and β for the following main reasons:

1. Definitional domain match: The Beta distribution is defined in the interval [0, 1], which fits the range of values of the adaptive parameters α and β. By limiting the range of α and β, it can ensure the stability of the noise covariance adjustment and avoid the parameter exceeding the reasonable range leading to numerical dispersion.
2. Flexibility: The shape of the Beta distribution is controlled by hyperparameters α and β, allowing flexibility in expressing different prior beliefs.
3. Regularization: The Beta distribution is able to avoid extreme values of α and β by adjusting the shape parameter, while the parameter design makes α and β tend to intermediate values, balancing the weights of historical information and current observations.
4. Computational convenience: The Beta distribution is the conjugate prior of the binomial distribution. Although the likelihood function of observation noise in BAEKF is the Gaussian distribution, the analytical nature of the Beta distribution can still simplify the process of updating the posterior distribution. Meanwhile, combined with the maximum a posteriori estimation (MAP), the optimal parameters can be solved effectively.

Second, the likelihood function is calculated based on the observed data, assuming that the observed data $S_{w}$ and $S_{v}$ follow a Gaussian distribution. The likelihood function can be defined as follows:

(34) $$p\left(S_{w}|\alpha \right)=N\left(S_{w};Q_{k-1},\alpha \right)$$

(35) $$p\left(S_{v}|\beta \right)=N\left(S_{v};R_{k-1},\beta \right)$$

Subsequently, the posterior distributions of α and β are updated using Bayesian reasoning, combining the prior distribution and the likelihood function to calculate the posterior distribution. The specific calculation formula is as follows:

(36) $$p\left(\alpha |S_{w}\right)\propto p\left(S_{w}|\alpha \right)p\left(\alpha \right)=N\left(S_{w};Q_{k-1},\alpha \right)\cdot Beta\left(\alpha ;a\alpha ,b\alpha \right)$$

(37) $$p\left(\beta |S_{v}\right)\propto p\left(S_{v}|\beta \right)p\left(\beta \right)=N\left(S_{v};R_{k-1},\beta \right)\cdot Beta\left(\beta ;a\beta ,b\beta \right)$$

Ultimately, the optimal values of the adaptive tuning parameters α and β are determined by maximum a posteriori (MAP) estimation. The optimum value allows for more accurate updating of the process noise covariance matrix $Q_{k}$ and the observation noise covariance matrix $R_{k}$. Accurate noise estimation helps to improve the accuracy of the calculation of the Kalman gain $K_{k}$, as well as the updating of the state estimate ${\hat{X}}_{\left(k|k\right)}$ and the covariance matrix $P_{\left(k|k\right)}$.

(38) $$K_{k}=P_{k|k-1}H_{k}^{T}{\left(H_{k}P_{k|k-1}H_{k}^{T}+R_{k}\right)}^{-1}$$

### 5.2. Adaptive Updating Steps

By improving the conventional EKF by introducing an adaptive method in its update step, the process noise covariance matrix is dynamically adjusted by combining the latest process noise estimation and the historical process noise covariance matrix in order to improve the adaptive ability and robustness of the filter under a change in noise environment. The adaptive approach utilizes weighted averaging and real-time error updating strategies to adaptively adjust the process noise covariance matrix $Q_{k}$ and the observation noise covariance matrix $R_{k}$ in order to optimize the estimation of the noise based on the observed data and prediction errors at each moment in time. The specific calculation formula is as follows:

#### 5.2.1. Estimation of Process Noise

(39) $$\left.\left\{\begin{array}{c} {\hat{w}}_{k}=X_{k}-F_{k-1}{\hat{X}}_{k|k-1}\\ S_{w}={\hat{w}}_{k}{\hat{w}}_{k}^{T}\\ Q_{k}=\alpha Q_{k-1}+\left(1-\alpha \right)S_{w} \end{array}\right.\right.$$

where ${\hat{w}}_{k}$ is the process noise estimate, which is calculated by the difference between the actual state and the predicted state, reflecting the effect of the model dynamic error on the system state. $S_{w}$ is the covariance of the prediction error and $S_{v}$ is the covariance of the observation error. The covariance matrix reflects the uncertainty of the noise source and affects the noise updating process. α is an adaptive tuning parameter in the weighted average approach to smooth the update process noise covariance matrix. When there is a sudden increase in process noise, $S_{w}$ increases and the BAEKF automatically reduces the α weights so that $Q_{k}$ responds quickly to the noise change. By smoothing the update, historical information can be retained while taking into account the most recent observations, allowing the process noise covariance matrix to be dynamically adjusted to changing circumstances.

#### 5.2.2. Estimation of Observation Noise

(40) $$\left.\left\{\begin{array}{c} {\hat{v}}_{k}=Z_{k}-H_{k}{\hat{X}}_{k|k-1}\\ S_{v}={\hat{v}}_{k}{\hat{v}}_{k}^{T}\\ R_{k}=\beta R_{k-1}+\left(1-\beta \right)S_{v} \end{array}\right.\right.$$

where ${\hat{v}}_{k}$ is the observation noise estimate that represents the difference between the observed and predicted observations. $S_{v}$ is the covariance of the observation error. β, as α, is the adaptive tuning parameter in the method of weighted averaging. When there is a sudden increase in observation noise, $S_{v}$ increases, and BAEKF automatically reduces the β weights so that $R_{k}$ quickly adapts to the observation error. Through Bayesian inference, combining the a priori information and real-time observation data, these parameters are dynamically adjusted so that the system can be more accurate in dealing with uncertainties in complex environments.

#### 5.2.3. Covariance Update

The formula for the covariance update is shown below:

(41) $$P_{k|k}=\left(I-K_{k}H_{k}\right)P_{k|k-1}$$

where $P_{k|k}$ denotes the state estimation covariance matrix at the current moment, reflecting the uncertainty of the state estimation. A smaller covariance indicates a more accurate state estimate. The covariance update formula corrects the predicted covariance matrix $P_{k|k-1}$ by the Kalman gain $K_{k}$ to obtain the updated covariance matrix $P_{k|k}$. In satellite orbit determination, if the quality of the observed data is higher ($R_{k}$ is smaller), $K_{k}H_{k}$ is larger and the covariance matrix $P_{k|k}$ is significantly smaller, indicating a lower uncertainty in the state estimation; if the quality of the observed data is poorer ($R_{k}$ is larger), then $K_{k}H_{k}$ is smaller and the covariance matrix $P_{k|k}$ is less variable, indicating that the uncertainty in the state estimate is still high.

#### 5.2.4. Status Update

State and covariance updating is the core step of BAEKF, where the predicted state is fused with the observed data through Kalman gain to obtain a more accurate state estimation and to update the uncertainty of the state. The specific formula is as follows:

(42) $${\hat{X}}_{k|k}={\hat{X}}_{k|k-1}+K_{k}\left(Z_{k}-H_{k}{\hat{X}}_{k|k-1}\right)$$

where ${\hat{X}}_{k|k}$ denotes the optimal state estimate at the current moment corrected for the observed data. $H_{k}{\hat{X}}_{k|k-1}$ denotes the observation residual, which responds to the deviation between predicted and actual observations and is used to correct the predicted state. A more accurate state estimate is obtained by weighting the observation residuals into the predicted state via Kalman gain $K_{k}$. The state update is based on the observation residuals. If the observation residuals are small, the state update relies mainly on the prediction model; if the observation residuals are large, the state update relies more on the observed data.

## 6. Analysis of Experiments and Results

### 6.1. Experimental Data

The astronomical optical observations used in this study were obtained from the Weihai Observatory of the National Astronomical Observatory of the Chinese Academy of Sciences. The Weihai Observatory is located in Weihai City, Shandong Province, China, at 121° E longitude and 37° N latitude, and its geographic location provides excellent observing conditions. The Weihai Observatory used the 2.5 m aperture Tian Xuan telescope to make short-arc optical observations of several satellite targets at different time periods, providing a large number of data for this experiment.

The study used space-based telescopes to obtain information on the orientation and elevation angles of different targets in several separate observation missions at different times. Table 1 shows some of the data from one of the observation missions. A follow-up comparison with the known TLE database shows that the observed targets for the presented data are “ONEWEB-0547”.

Preprocessing of the loaded data is required in the algorithm, mainly to convert the raw observations into a form suitable for use by the filter and to compute some necessary intermediate variables. The following is a detailed description of the logarithmic preprocessing. First, the date and time in the loaded data need to be merged to form a complete UT timestamp for subsequent time calculations. Second, the location (latitude and longitude elevation, LLE) and error of the observatory are set, as well as the time of the initial trial and the reference orbit for the subsequent calculation of the observation noise covariance matrix R. Third, based on the time and LLE, the position of the observatory in the ECI coordinate system is calculated, while the azimuth and elevation angles are converted to right ascension and declination, and combined with the distance to compute the apparent distance vector in ECI. The line-of-sight vector is added to the observatory position to obtain the position of the target in the ECI. Fourth, multiple sets of positions and velocities are averaged to generate the nominal state, and the initial state is obtained by propagating the nominal state to the initial time. Finally, the observations are combined and the noise covariance is calculated based on the error.

### 6.2. Experimental Results

**Figure 3:**
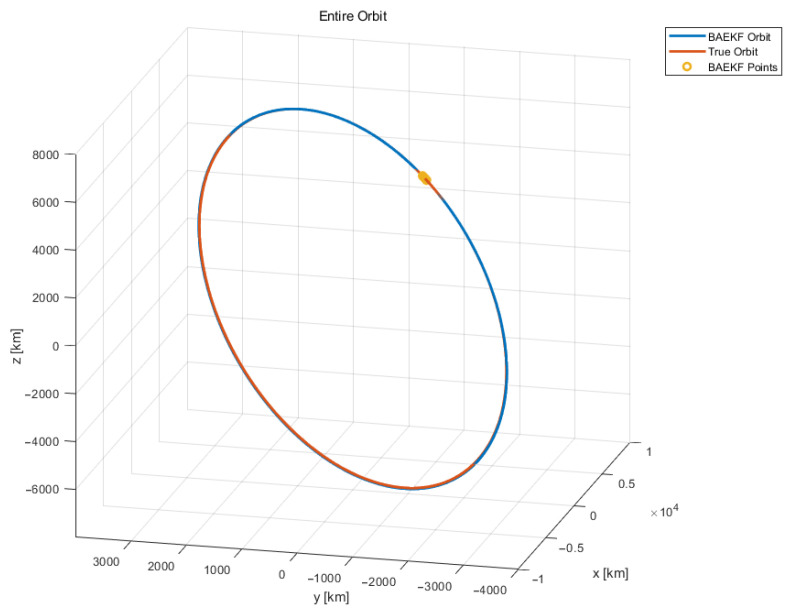
Overall optical observation orbit determination.

**Figure 4:**
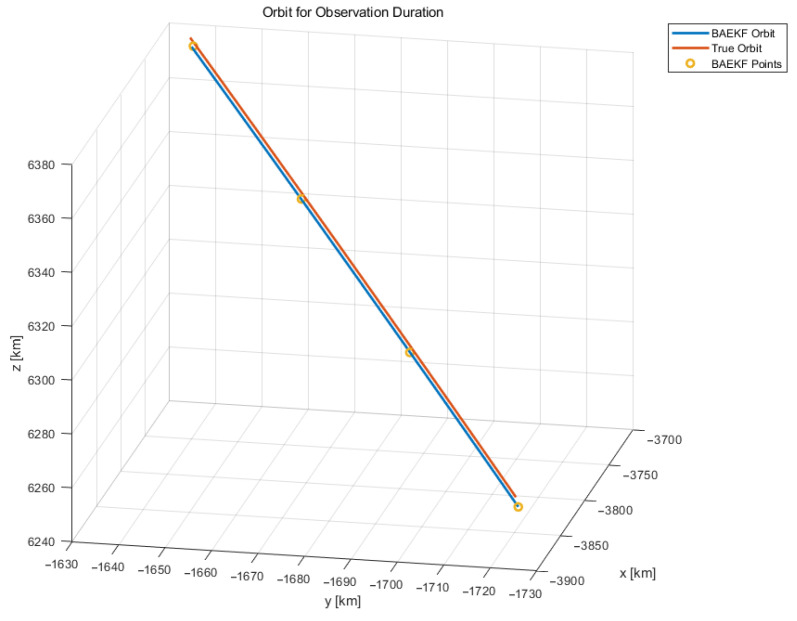
Localized map for optical observation orbit determination.

The experiment begins with denoising and removing outliers from the initial angular observation data to ensure the reliability and accuracy of the data. Subsequently, the azimuth and elevation observations at different moments are used to compute the current moment’s right ascension and declination coordinate data, respectively, which can be used for the subsequent computation of the position vector and velocity vector. The experiment uses an Adaptive Extended Kalman Filter introducing Bayesian reasoning to compute the calculated position vectors and velocity vectors at each moment in time for orbit determination. The satellite orbit maps obtained from Bayesian-based Adaptive Extended Kalman Filter estimation are shown in Figure 3 and Figure 4. The overall image of the predicted and true orbits of the observed target is shown in Figure 3, and the overall image of the local predicted and true orbits of the observed target at some of the observed moments is shown in Figure 4.

In Figure 4, the orange trajectory line is the real satellite orbit resolved by using TLE data, the yellow trajectory circle indicates the position coordinate point at a certain moment calculated by using the observation angle data at that moment, and the blue trajectory line is the predicted satellite orbit obtained by estimating the BAEKF method using a limited number of position coordinate points. In Figure 4, the real satellite orbit and the predicted satellite orbit have trajectories that partly overlap, or the two orbits are obscured from each other. The TLE data of the observation target ONEWEB-0547, which was validated in the experimental follow-up, were among the data used (Table 2).

### 6.3. Experimental Analysis

#### 6.3.1. Comparison of Classical Differential Correction Algorithms

Classical differential correction algorithms are a family of methods that use the idea of differential correction to optimize orbital parameters in orbit determination tasks. The core of these methods is based on the nonlinear least squares method to minimize the residuals between predicted and actual observations by iteratively differentially correcting the orbit parameters. Classical differential correction algorithms were widely used in the mid-20th century in the field of aerospace engineering because of their simplicity and computational feasibility. Comparing the batch orbit determination method to the modern sequential filtering method, there are limitations in real-time, dynamic noise adaptability, and nonlinear processing capability, which are mainly reflected in the following aspects:

1. Algorithm types: Classical differential correction algorithms are a class of batch algorithms that emphasize global optimization and are suitable for non-real-time tasks; BAEKF is a sequential filtering method that emphasizes real-time and is capable of dynamically updating the state estimates as new observations arrive.
2. Dynamic noise adaptation: Classical differential correction algorithms assume that the statistical characteristics of noise are fixed, making it difficult to cope with dynamic noise; BAEKF adapts to noise fluctuations in real-time through adaptive noise covariance adjustment, reducing the impact of abnormal data.
3. Nonlinearity handling capability: Classical differential correction algorithms deal with nonlinearities through local linearization, ignoring higher-order terms, which may lead to error accumulation; BAEKF introduces Bayesian a posteriori probability corrections to compensate for higher-order nonlinear terms and reduce errors in long-term predictions.

#### 6.3.2. Comparing Modern Algorithms

**Figure 5:**
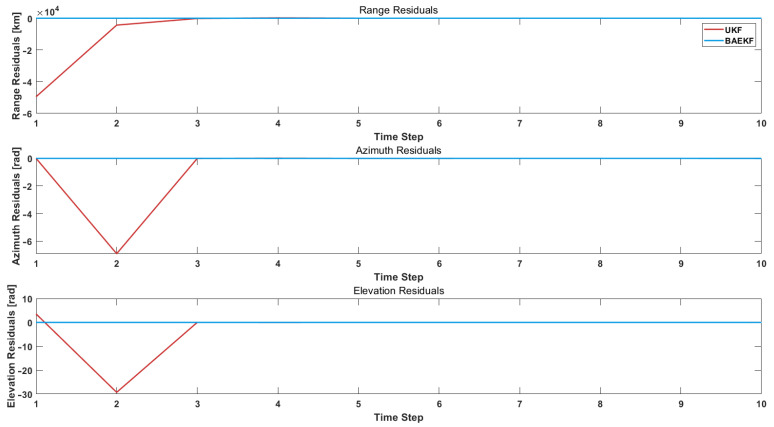
Residual images of BAEKF method and UKF method in predicting the same orbit.

In order to measure the prediction accuracy of the BAEKF method from a global perspective, the experiment first used the residual and RMSE as metrics to evaluate the performance of the filter; Mean Absolute Error (MAE) was also added as a secondary indicator in Table 3. The residual is the difference between the observed and predicted values. By analyzing the residuals, the instantaneous error performance of the filter can be evaluated at each time step, which helps to identify transient error hotspots and monitor the performance of the filter at a specific moment in time. In this paper, the improved BAEKF algorithm is used with the traditional EKF, UKF, RBFNN, and GPR methods to compute the residuals for range, azimuth, and elevation, respectively. The specific formulas for the residuals are as follows:

(43) $$\epsilon_{i}=x_{i}-{\hat{x}}_{i}$$

where $x_{i}$ is the observed value at time step *i* and $\hat{x_{i}}$ is the predicted value at time step *i*. In this paper, BAEKF is compared with traditional UKF for individual residuals because the residual values of traditional UKF need many iterations to converge and the residual values are very large in the initial stage. Figure 5 shows the residual images of the observed target orbit prediction using the BAEKF method and UKF method.

**Figure 6:**
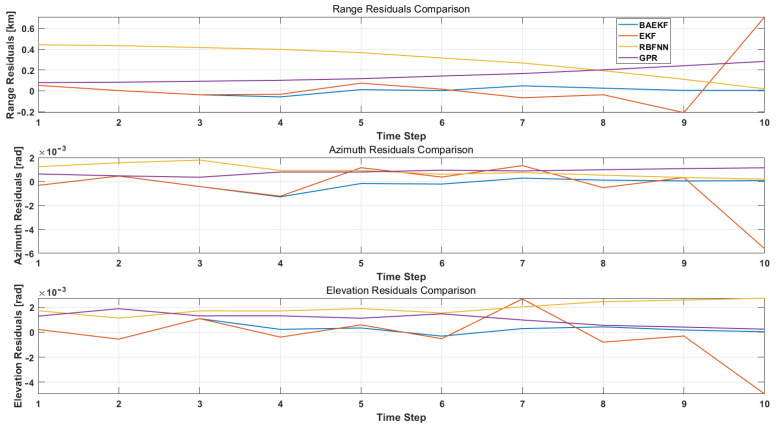
Residual images of BAEKF method and other methods in predicting the same orbit.

Traditional UKF algorithms represent state distributions via Sigma point sets rather than point estimates or linearized approximations. This representation, while capturing a more complete characterization of the nonlinear system, displays a larger range of estimation bias in the initial phase. As in Figure 5, the range residuals differ by four orders of magnitude at the initial stage. And UKF uses the Unscented transform to propagate the entire probability distribution directly through the nonlinear system rather than relying on a linearized approximation, resulting in an initial stage that will show the nonlinear effects of the system more fully. In order to be able to visualize the variation of the residuals of the four algorithms in a more intuitive way, the residuals of the BAEKF versus the EKF, RBFNN, and GPR algorithms are plotted in Figure 6.

Range residuals reflect the algorithm’s error performance in estimating the distance between the satellite and the observatory, and are a key measure of orbit determination accuracy. In Figure 6, the distance residuals of the EKF fluctuate widely, especially during time steps 5–10, and the residual values oscillate drastically between −0.2 km and 0.4 km, indicating that the EKF is not sufficiently adapted to the nonlinear dynamical model. The main reason is that the EKF relies on the first-order Taylor expansion linearization and ignores the higher-order nonlinear terms, which leads to the accumulation of the linearization error over time, which is significantly magnified especially in the case of sparse short-arc observations. The fluctuation of distance residuals of RBFNN is slightly smaller than that of EKF, but it still exhibits large deviations at time steps 8–10. It mainly captures nonlinear perturbations through radial basis function neural networks, but its performance is highly dependent on the training quality of historical data. In the face of sudden dynamic noise, RBFNN has difficulty adapting quickly, resulting in large fluctuations in the residuals, and the fluctuations in the distance residuals of GPR are comparable to those of RBFNN, with a large deviation at time steps 8–10. GPR models nonlinear systems through Gaussian process regression, but it is sensitive to track geometry, and the computational complexity increases exponentially with the increase in data volume, making it difficult to converge stably under sparse data conditions. GPR models the nonlinear system by Gaussian process regression.

Analyzed in terms of azimuth residuals, the azimuth residuals of the EKF show periodic shifts. Moreover, since the EKF assumes fixed noise statistics, it is difficult to adapt to the dynamic angular jitter caused by atmospheric turbulence in optical observation, which leads to the amplification of systematic bias, and the maximum residuals can reach −0.006 rad. The fluctuation of azimuth residuals of RBFNN is slightly smaller than that of EKF, but it still exhibits a large deviation. RBFNN learns nonlinear dynamics through a neural network, but its training process relies heavily on historical data, and it has difficulty converging quickly under the short-arc data condition, which leads to a large error in azimuth estimation. The maximum residual value of the azimuthal residuals of GPR is approximately 0.001 rad. GPR is more sensitive to the orbit geometry, and the uncertainty of the initial state tends to lead to the accumulation of the bias of the azimuthal estimation under the short-arc observations. The fluctuation of the azimuthal residuals of BAEKF is significantly reduced and stays in the range of −0.001 to 0.0005 rad. By incorporating geometric constraints from multimoment angular observations, the BAEKF enhances the visualizability of the azimuthal parameters, which converges quickly even in the early stage of data sparsity and avoids the amplification of systematic biases. In addition, the Bayesian framework dynamically corrects the posterior probability distribution of the state estimation, further suppressing the nonlinear error.

In terms of elevation residuals analysis, the EKF’s elevation residuals gradually accumulate with time steps and reach a maximum value at time steps 8–10, indicating that its linearized model is inadequate for modeling long-term orbital uptake. The EKF ignores the higher-order nonlinear terms, which leads to the accumulation of errors over time, and the bias is especially significant in long-period forecasting. The fluctuation amplitude of elevation angle residuals of RBFNN is significantly smaller than that of the EKF. Although RBFNN can capture part of the nonlinear perturbations, it lacks a dynamic noise adjustment mechanism in the face of long-term uptake, which leads to a gradual increase in the elevation angle estimation error. The fluctuation of the elevation residuals of GPR is close to that of RBFNN. The computational volume of GPR grows exponentially with the amount of data, and it is difficult to maintain the stability in long-period prediction, which leads to the accumulation of elevation estimation errors. The fluctuation of elevation angle residuals of BAEKF is minimized, which is maintained between −0.0005 rad and 0.0015 rad. BAEKF dynamically corrects the effect of higher-order nonlinear terms through the Bayesian framework, while the adaptive noise covariance adjustment mechanism effectively suppresses the observation jitter, so that the residuals do not show an obvious accumulation trend.

The adaptive noise covariance adjustment mechanism of BAEKF can respond to dynamic noise in real-time and keep the residuals stabilized when the noise changes abruptly. Under short-arc data conditions, BAEKF enhances the visualization of orbit parameters by fusing geometric constraints from multi-moment angular observations, especially in azimuth and elevation estimation, which can converge quickly and avoid the high sensitivity of EKF and GPR to initial errors. Compared with RBFNN and GPR, BAEKF does not rely on a large amount of historical data for training and has lower computational complexity.

The Root Mean Square Error (RMSE) is the square of the residuals and the square root of the mean, and measures the cumulative value of the error in terms of the overall time period, reflecting the overall prediction accuracy. The RMSE is more sensitive to larger errors than the mean error (ME) alone. Figure 6 shows the RMSE images of the orbital prediction of the observed target using the BAEKF method and the EKF method. The specific calculation formula is as follows:

(44) $$RMSE=\sqrt{\frac{1}{n}\sum_{i=1}^{n}\left[{\left(x_{i}-{\hat{x}}_{i}\right)}^{2}+{\left(y_{i}-{\hat{y}}_{i}\right)}^{2}+{\left(z_{i}-{\hat{z}}_{i}\right)}^{2}\right]}$$

The reasons for the fluctuations in the RMSE may be multiple. First, optical angle measurements are disturbed by atmospheric turbulence, equipment thermal noise, and other factors, resulting in jittery azimuth and elevation angle data, and inaccurate measurements received by the system can lead to fluctuations in the filter output error. Second, the higher-order uptake forces neglected in the dynamical model introduce systematic bias in the long-term prediction. The initial orbital parameter estimation errors under the short-arc data are gradually amplified with filter iterations.

**Figure 7:**
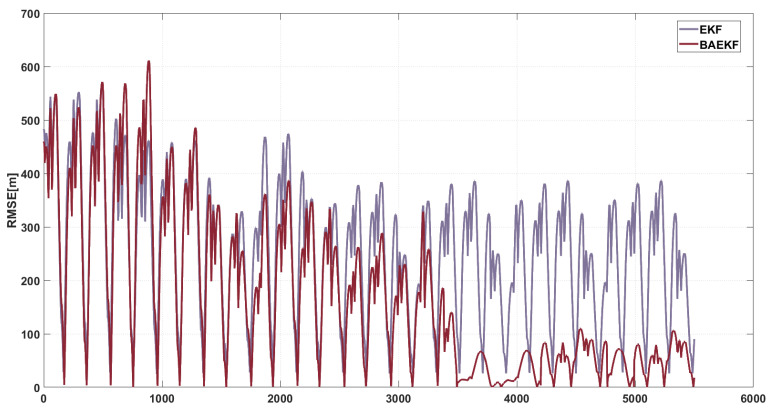
RMSE images of BAEKF method and EKF method in predicting same orbit.

As shown in Figure 7, the RMSE of the EKF peaks during the time steps 0–2000, then gradually decreases in the time steps 2000–4000, and then fluctuates significantly in the time steps 4000–6000, with the maximum value close to 400 m. The average RMSE value of the EKF during the whole orbiting period is about 184.82 m. Due to the fact that the EKF is linearized by the first-order Taylor expansion of the nonlinear term, the linearization error is higher and accumulates significantly with time. Since the EKF ignores the higher-order nonlinear terms through the first-order Taylor expansion, the linearization error accumulates with time, and the overall RMSE of the EKF is high and shows an obvious accumulation trend. It is highly sensitive to the initial error under short-arc data conditions, and the error propagation is further amplified, making it difficult to adapt to the dynamically changing noise in the optical observation.

**Figure 8:**
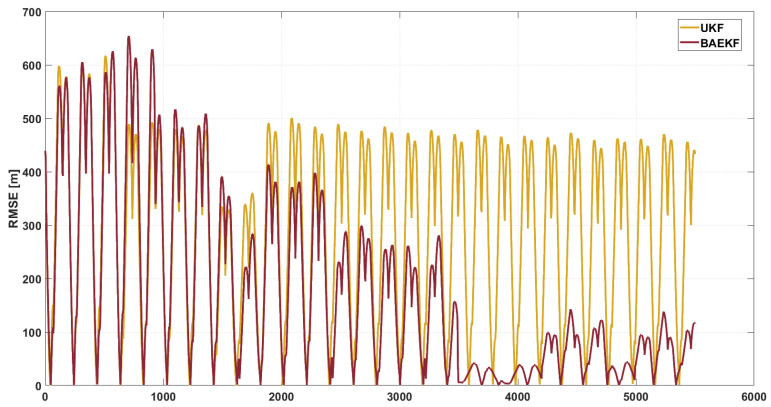
RMSE images of BAEKF method and UKF method in predicting same orbit.

In Figure 8, the RMSE of UKF peaks during time steps 0–1000. A large fluctuation occurs during the time step 1000–2000. Subsequently, during the time step 2000–6000, the RMSE value remains relatively stable but still at a high RMSE value around 500 m. The average RMSE value of UKF during the whole orbiting period is about 194.14 m. UKF approximates the nonlinear distribution by the unscented transformation, which reduces the linearization error compared with EKF, but its computational complexity grows cubically with the state dimension, which makes it difficult to meet the real-time requirement. At the same time, UKF assumes that the statistical characteristics of noise are fixed, which generates difficulties in adapting to the dynamic noise environment, resulting in large error fluctuations.

**Figure 9:**
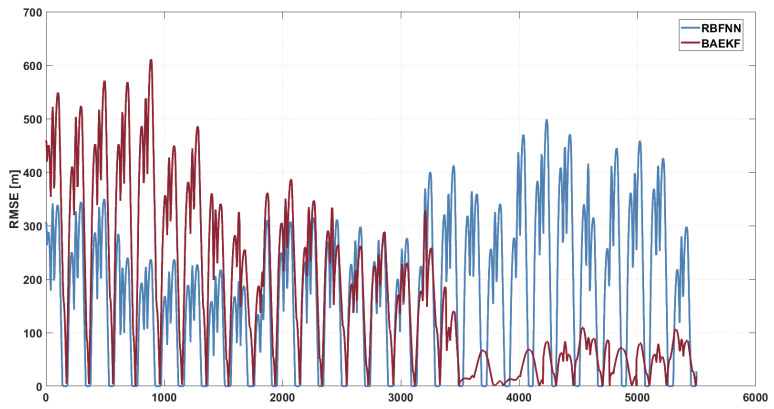
RMSE images of BAEKF method and RBFNN method in predicting same orbit.

As shown in Figure 9, the RMSE of the RBFNN exhibits large fluctuations throughout the time period, especially during the time step 2000–5000, when the RMSE value reaches as high as 500 m. The average RMSE value of the RBFNN during the whole period of orbiting is about 163.87 m. The average RMSE value of the RBFNN during the whole period of orbiting is about 163.87 m. Such large fluctuations indicate the instability of RBFNN in dealing with nonlinear dynamics and dynamic noise. RBFNN relies on radial basis function neural networks to capture nonlinear perturbations, and its performance is highly dependent on the training quality of historical data. Under the condition of sparse short-arc observation data, it is difficult for RBFNN to quickly adapt to dynamic noise, leading to error accumulation and increased fluctuation. In addition, the high computational complexity of RBFNN makes it difficult to maintain stability in long-time prediction, leading to maintaining high RMSE values in subsequent time steps.

**Figure 10:**
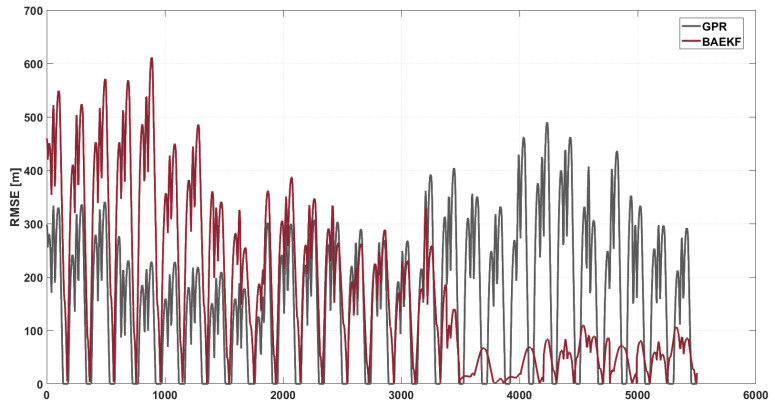
RMSE images of BAEKF method and GPR method in predicting same orbit.

In Figure 10, the RMSE of GPR is maintained between 200 and 300 m during the time step 0–3000, but during the same time, there are large fluctuations. With the accumulation of errors and the sudden change of noise, it reaches the peak value during the time step 4000–5000, and the RMSE of GPR again shows large fluctuations, with the maximum value close to 500 m. The RMSE of GPR is also shown in Figure 10, which is the same as the RMSE of GPR. The average RMSE value of GPR during the whole orbit fixing period is about 156.48 m. GPR models the nonlinear system by Gaussian process regression, but it is highly sensitive to the orbit geometry, and the uncertainty of the initial state under the short-arc data conditions tends to lead to the accumulation of errors. In addition, the computational complexity of GPR grows exponentially with the number of data, which makes it difficult to maintain the stability in long-time prediction, especially when facing dynamic noise.

In contrast, the RMSE fluctuation of BAEKF is significantly reduced, and the RMSE value gradually decreases from a large fluctuation and stays at a low level. During the time step 4000–5000 noise mutation, the RMSE of other algorithms generally fluctuates greatly, while the RMSE of BAEKF only increases slightly. The average RMSE value of GPR during the whole orbiting period is about 120.68 m. Compared with the first-order linearization or the traceless transform of EKF and UKF, BAEKF compensates for the higher-order nonlinear terms better, and reduces the error in the long-term prediction significantly. Compared with the high computational complexity of RBFNN and GPR, and the cubic growth of UKF, BAEKF has lower computational complexity, and realizes efficient state estimation while maintaining high accuracy. Table 3 visualizes the RMSE values of each algorithm. Meanwhile, MAE and overall residual fluctuation are added as indicators in the table to further highlight the performance of different algorithms; ‘-’ denotes the algorithm’s decreased accuracy compared to BAEKF.

**Table 3:** Comparison of RMSE and MAE of different algorithms.

Algorithms	Average RMSE	RMSE Accuracy	Average MAE	MAE Accuracy	Residual Fluctuation
BAEKF	120.6758	1	60.3137	Data	1
EKF	184.8235	−34.71%	108.021	−44.16%	−40.5%
UKF	194.142	−37.83%	115.4829	−47.76%	–
RBFNN	163.8711	−26.37%	96.6842	−37.61%	−32.6%
GPR	156.4762	−22.875%	92.3992	−34.72%	−30.4%

## 7. Conclusions

For optical telescope-acquired satellite angular observation data, this study develops a Bayesian Adaptive Extended Kalman Filter (BAEKF)-based orbit determination methodology for optical observation satellites. The primary innovation lies in substantially enhancing the robustness and accuracy of conventional Extended Kalman Filters through a dual-mechanism approach combining dynamic noise covariance adaptation and Bayesian posterior probability correction. By employing optical angular observation data to compute target position and velocity parameters, BAEKF enables real-time optimization of noise covariance matrices $Q_{k}$ and $R_{k}$ through Bayesian inference integration and exponentially weighted moving averaging. The experimental results show that, compared with the traditional EKF, UKF, RBFNN, and GPR methods, BAEKF demonstrates better stability while maintaining lower residual values, and its residual fluctuation is significantly reduced, which reflects the robustness of the algorithm in dynamic noise environments. In terms of the global performance of RMSE, BAEKF reduces the RMSE by 34.71% compared with EKF, 37.83% compared with UKF, 26.37% compared with RBFNN, and 22.875% compared with GPR, and it not only has the advantage of accuracy in the processing of nonlinear systems and sparse data, but also has the advantage of accuracy in the processing of nonlinear systems and sparse data through adaptive noise, covariance adjustment, and Bayesian noise. BAEKF not only has the advantage of accuracy when dealing with a nonlinear system and sparse data, but also can overcome the limitations of EKF and UKF under the fixed assumption of noise statistical characteristics, as well as the shortcomings of RBFNN and GPR regarding their dependence on historical data and computational complexity through adaptive noise covariance adjustment and its Bayesian a posteriori probability correction mechanism.

Although demonstrating BAEKF’s orbit determination superiority through comprehensive data analysis, several limitations warrant consideration. Future research should explore parallel computing architectures or hardware acceleration strategies to address computational complexity in Bayesian inference and adaptive tuning processes, thereby satisfying real-time orbit determination requirements. Additional verification is required for BAEKF’s applicability to HEO and NEO satellite scenarios, coupled with investigation of the impact of varying power system models on algorithmic performance.

## Figures and Tables

**Table 1:** Selected optical telescope observations.

Year Month Date	Time (UTC)	Azimuth (°)	Elevation (°)
2024 06 28	09: 16: 08.00	31.4286	6.3375
2024 06 28	09: 16: 18.00	32.4092	6.7375
2024 06 28	09: 16: 28.00	33.4119	7.1347
2024 06 28	09: 16: 38.00	34.4369	7.5292
2024 06 28	09: 16: 48.00	35.4250	6.3375
2024 06 28	09: 16: 58.00	36.4947	7.8980
2024 06 28	09: 17: 08.00	37.5875	8.2850
2024 06 28	09: 17: 18.00	38.7042	8.6673
2024 06 28	09: 17: 28.00	39.8444	9.0444
2024 06 28	09: 17: 38.00	41.0092	9.4158

**Table 2:** Spatial target TLE data.

ONEWEB-0547 TLE Data
1 56049U 23043D 25004.66144206 .00000238 00000+0 54545-3 0 9994
2 56049 87.9303 349.2857 0001740 91.7550 268.3783 13.21826925 88409

## Data Availability

The data supporting the findings of this study are available from the corresponding author upon reasonable request.
